# Supplementary figures and images for: Short-Chain Fatty Acid Production by Gut Microbiota from Children with Obesity Differs According to Prebiotic Choice and Bacterial Community Composition
Source: mBio. 2020 Aug 11;11(4):e00914-20. doi: 10.1128/mBio.00914-20 (PMC7439474; doi:10.1128/mBio.00914-20)

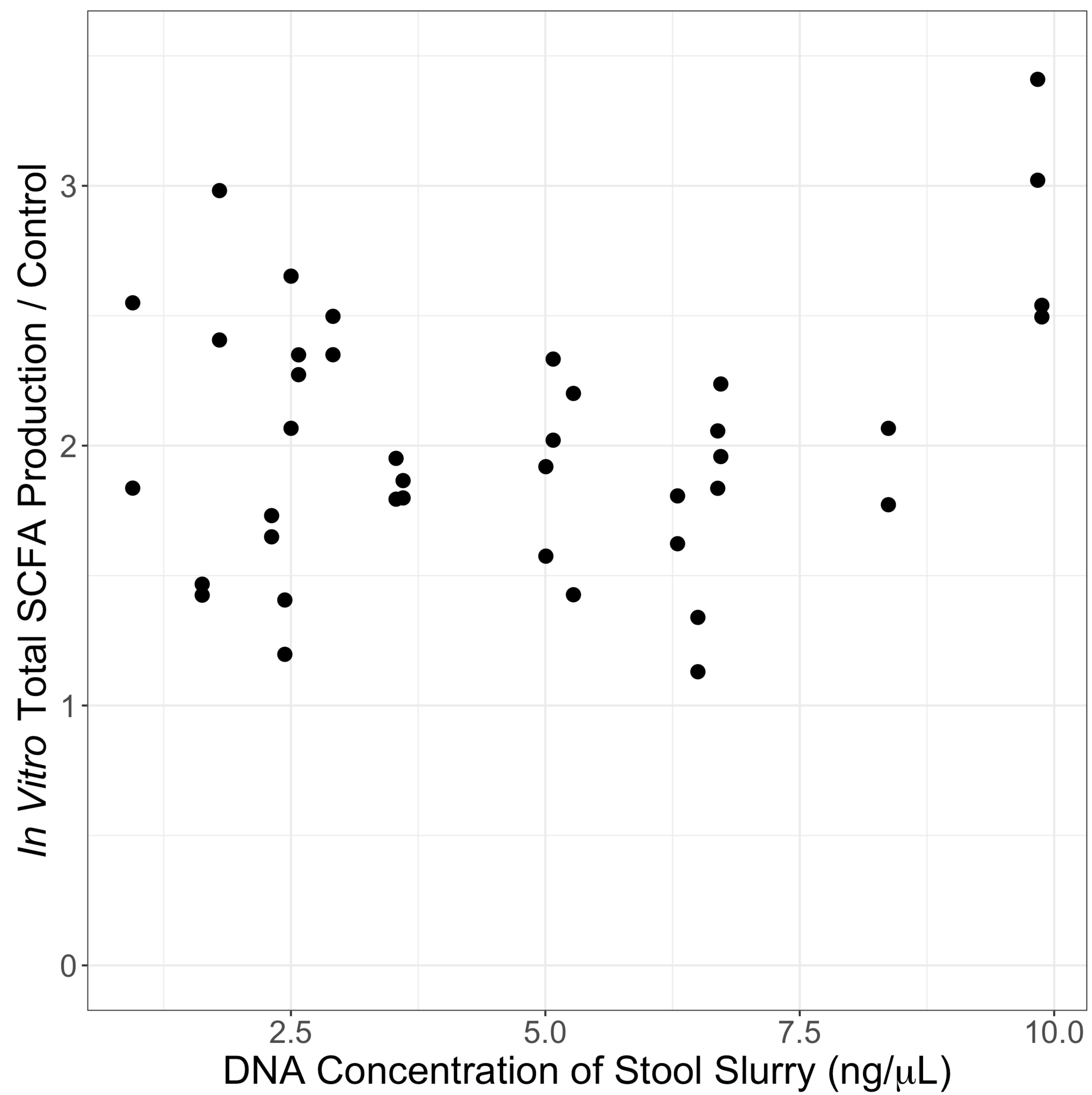

Supplement: FIG S1 [file mBio.00914-20-sf001.pdf]

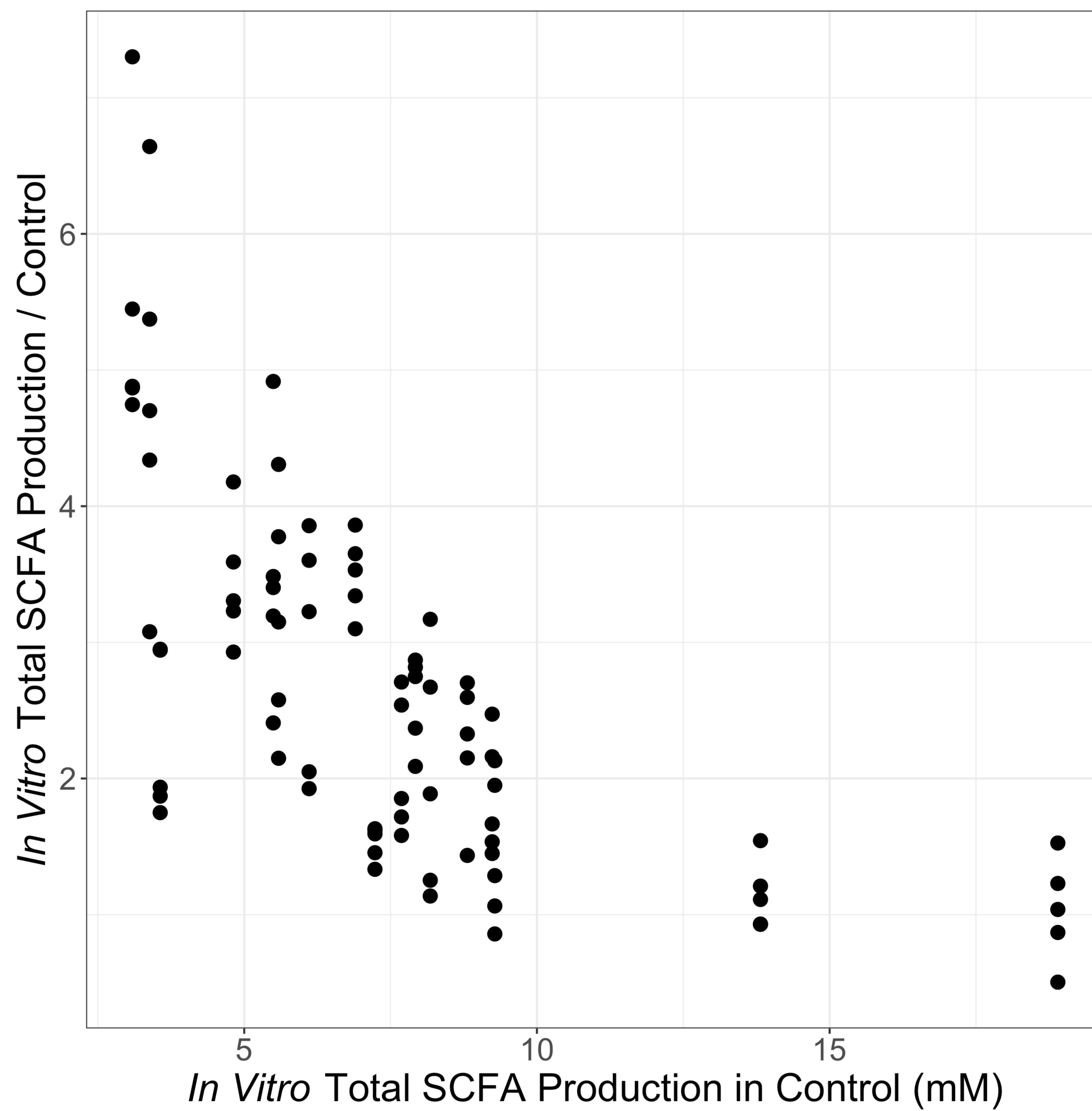

Supplement: FIG S2 [file mBio.00914-20-sf002.pdf]

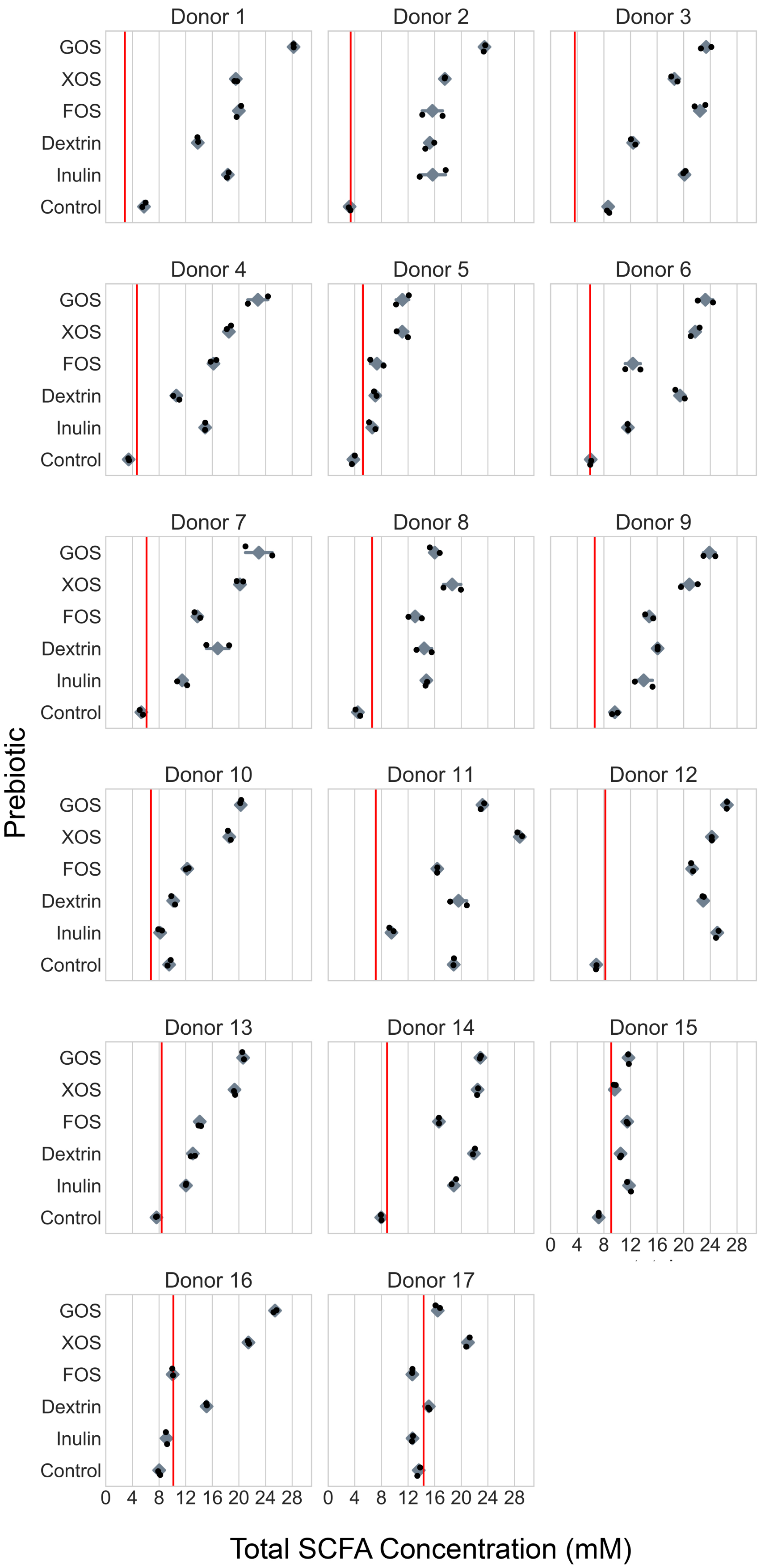

Supplement: FIG S3 [file mBio.00914-20-sf003.pdf]

A

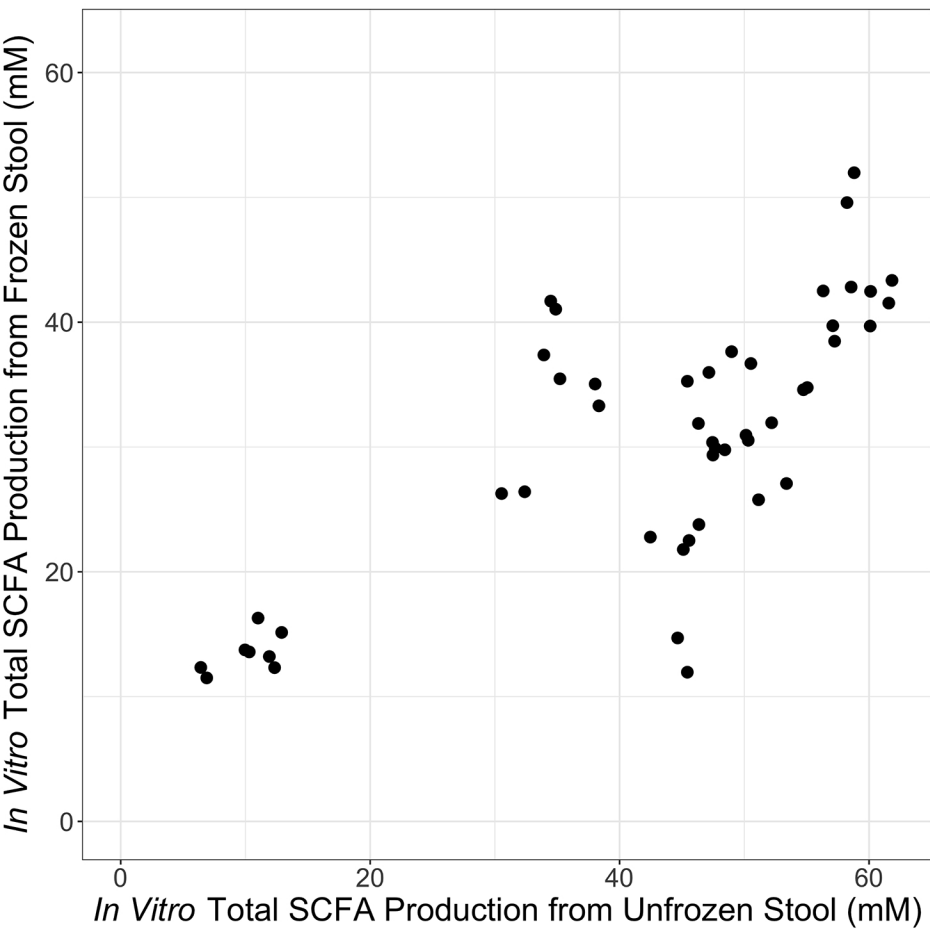

B

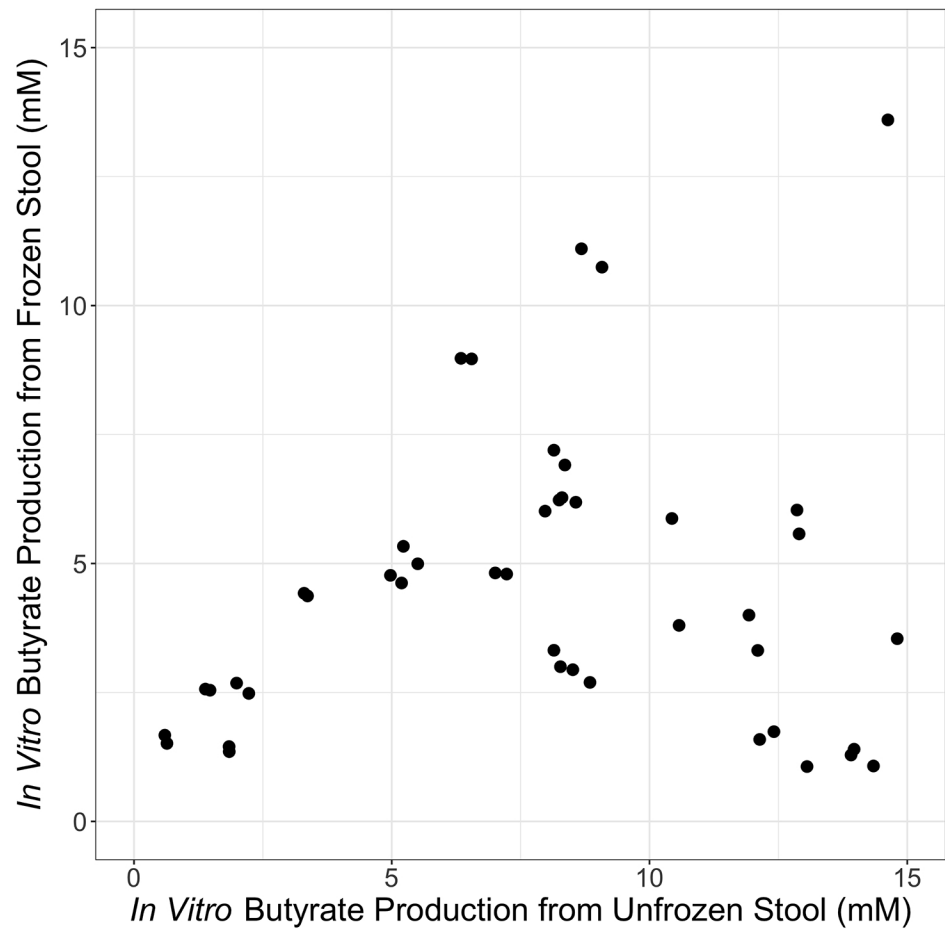

Supplement: FIG S4 [file mBio.00914-20-sf004.pdf]

A

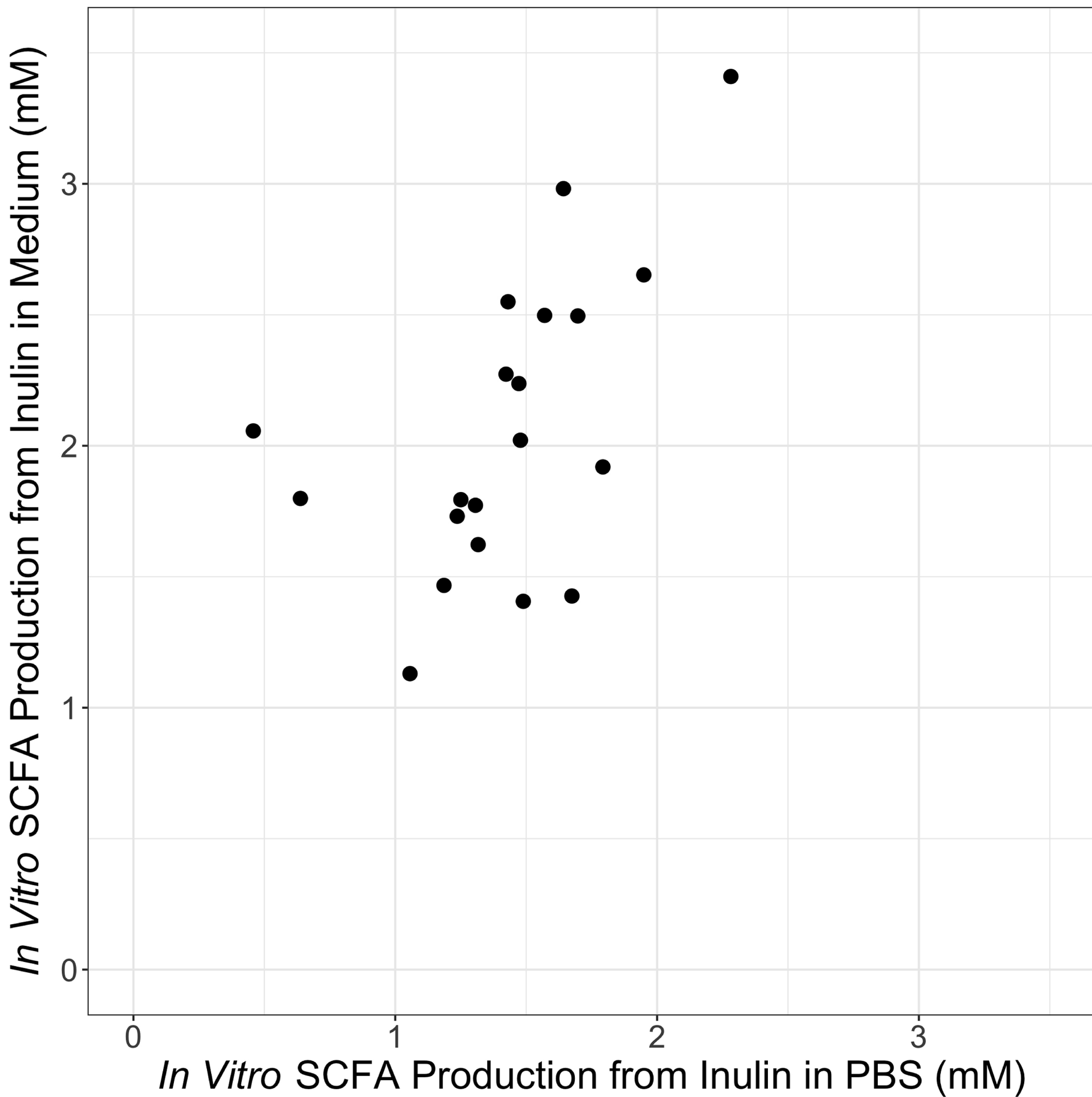

B

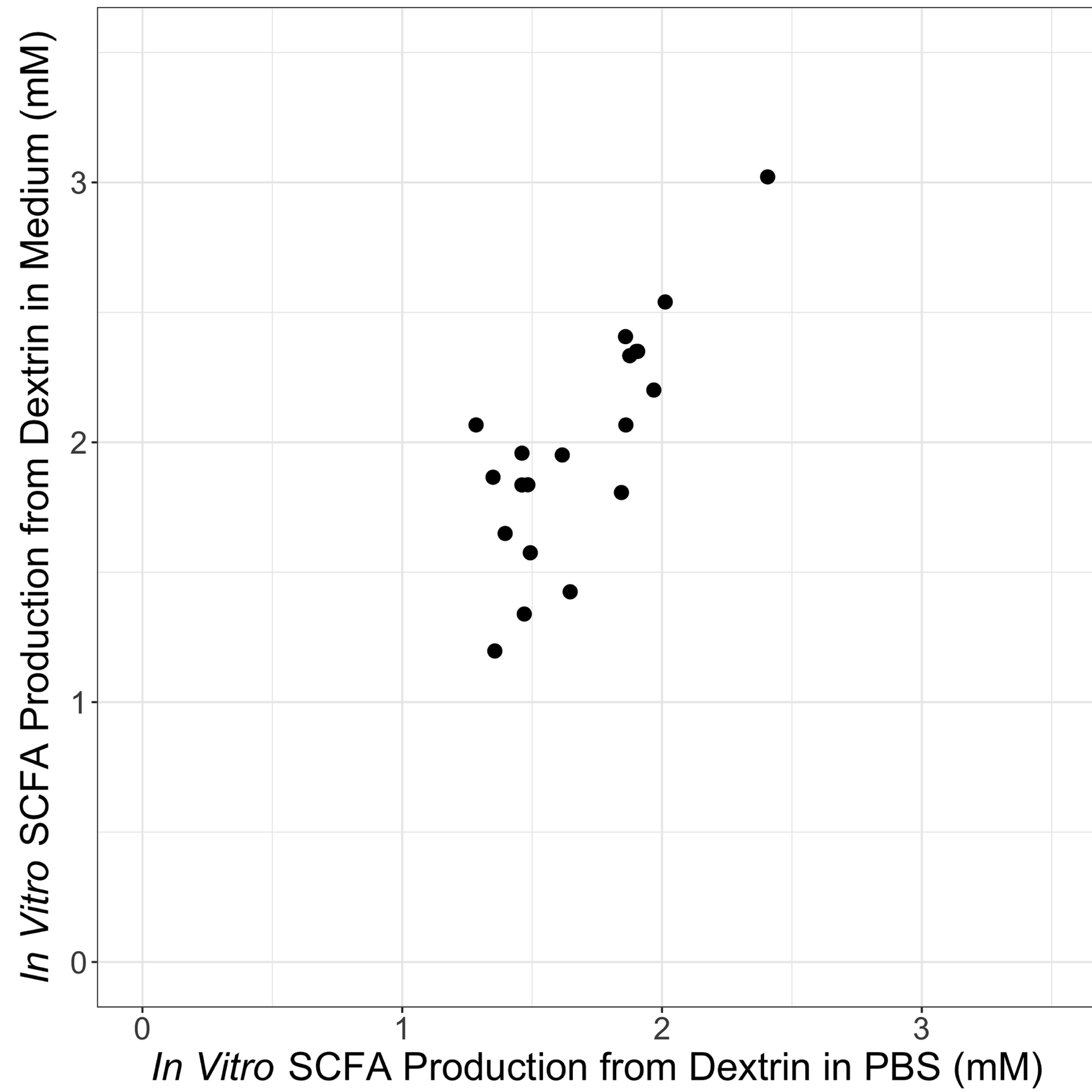

Supplement: FIG S5 [file mBio.00914-20-sf005.pdf]

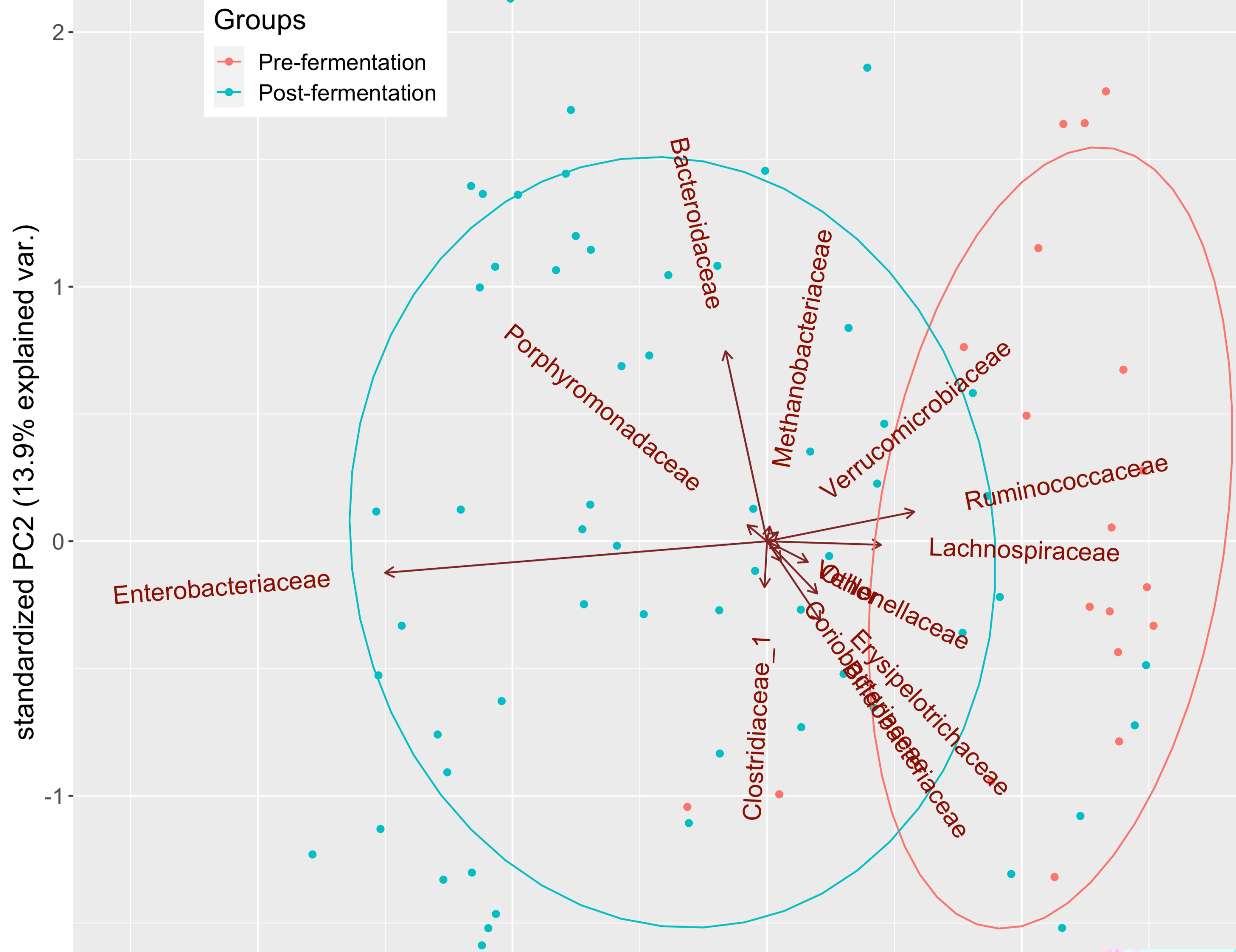

Supplement: FIG S6 [file mBio.00914-20-sf006.pdf]
