# Supplementary material for: Short-Chain Fatty Acid Production by Gut Microbiota from Children with Obesity Differs According to Prebiotic Choice and Bacterial Community Composition
Source: mBio. 2020 Aug 11;11(4):e00914-20. doi: 10.1128/mBio.00914-20 (PMC7439474; doi:10.1128/mBio.00914-20)
Supplement: TABLE S1 [file mBio.00914-20-st001.docx]

| Variable | Male (n) | Female (n) | BMI Range | Average BMI | Age Range | Average Age | Total |
| --- | --- | --- | --- | --- | --- | --- | --- |
| Value | 6 | 10 | 25.9 – 75.3 | 34.9 | 10-18 | 15.7 | 17 |
