# Supplementary material for: Short-Chain Fatty Acid Production by Gut Microbiota from Children with Obesity Differs According to Prebiotic Choice and Bacterial Community Composition
Source: mBio. 2020 Aug 11;11(4):e00914-20. doi: 10.1128/mBio.00914-20 (PMC7439474; doi:10.1128/mBio.00914-20)
Supplement: TABLE S2 [file mBio.00914-20-st002.docx]

| Source | *df* | *SS* | *F* | *p* |
| --- | --- | --- | --- | --- |
| Donor | 16 | 243.1 | 255.4 | <0.0001 |
| Prebiotic | 4 | 35.7 | 149.9 | <0.0001 |
| Donor*Prebiotic | 64 | 28.7 | 7.5 | <0.0001 |
