# Supplementary material for: Short-Chain Fatty Acid Production by Gut Microbiota from Children with Obesity Differs According to Prebiotic Choice and Bacterial Community Composition
Source: mBio. 2020 Aug 11;11(4):e00914-20. doi: 10.1128/mBio.00914-20 (PMC7439474; doi:10.1128/mBio.00914-20)
Supplement: TABLE S3 [file mBio.00914-20-st003.docx]

| Patient | Treatment | Timepoint (month) |
| --- | --- | --- |
| 1 | Lifestyle only | 6 |
| 2 | Lifestyle only | 0 |
| 3 | Lifestyle only | 3 |
| 4 | Lifestyle only | 0 |
| 5 | Lifestyle only | 6 |
| 6 | Lifestyle only | 4.5 |
| 7 | Lifestyle only | 0 |
| 8 | Lifestyle only | 3 |
| 9 | Lost to follow-up | 0 |
| 10 | Lifestyle only | 4.5 |
| 11 | Lifestyle only | 3 |
| 12 | Lifestyle only | 4.5 |
| 13 | Low carb | 0 |
| 14 | Lifestyle only | 4.5 |
| 15 | Metformin | 4.5 |
| 16 | Lifestyle only | 4.5 |
| 17 | Lifestyle only | 4.5 |
